# DOES AGE, RACE, AND/OR RURALITY DETERMINE OLDER VETERANS' ABILITY TO ACCESS VA VIDEO CONNECT

**DOI:** 10.1093/geroni/igac059.2230

**Published:** 2022-12-20

**Authors:** Linda Sawyer, Christine Cigolle, Kristin Phillips, Janette Dunlap, Dennis Sullivan

**Affiliations:** Central Arkansas VA Healthcare System, Sherwood, Arkansas, United States; VA Ann Arbor, Ann Arbor, Michigan, United States; Ann Arbor VA, Ann Arbor, Michigan, United States; North Florida South Georgia VA, Jacksonville, Florida, United States; Central Arkansas VA, North Little Rock, Arkansas, United States

## Abstract

The purpose of this project was to determine whether ability to use VA Video Connect (VVC) by older Veterans (OV) differed by age, race, or rurality. A service to help older Veterans learn to use VVC was developed. As part of an ongoing QI project, demographics, willingness to attempt a VVC test call and outcomes of test calls were collected on all referrals. Descriptive statistics, Chi-square, and Fisher’s exact test were used to examine differences in success rates by group. Of the 66 OV (age 60+) referred by their primary care providers, we were able to contact 63 by phone. Of those, 46 (73%) scheduled a VVC test call, 7 (11%) chose not to participate, and 10 (16%) were already using VVC for appointments. Of the 40 who continued the VVC test call, 31 (77.5%) were successful without issues, 7 (17.5%) were successful with help resolving issues, and 2 (5%) disconnected before finishing the call because it became too difficult. Of the 63 OV contacted, 38 (57.5%) had a successful VVC test call. However, those residing in rural (vs. urban) settings were less likely to have a successful test call (43% vs. 57%, p=0.04). There was no statistically significant difference in success rates for the test calls between whites vs. non-whites (52% vs. 48%, p= 0.2), or those aged 75 years or above vs. 60-74 years (53% vs. 46%, p=0.6). More work is needed to identify barriers to use of VVC, especially among OV living in rural settings.

